# 人工智能辅助诊断系统预测肺结节早期肺腺癌浸润亚型的临床研究

**DOI:** 10.3779/j.issn.1009-3419.2022.102.12

**Published:** 2022-04-20

**Authors:** 志鹏 苏, 文杰 毛, 斌 李, 智中 郑, 博 杨, 美玉 任, 铁牛 宋, 海明 冯, 于琪 孟

**Affiliations:** 730030 兰州，兰州大学第二医院胸外科，兰州大学第二临床医学院 Department of Thoracic Surgery, Lanzhou University Second Hospital, Lanzhou University Second Clinical Medical College, Lanzhou 730030, China

**Keywords:** 人工智能, 肺结节, 肺肿瘤, 腺癌, 浸润亚型, Artificial intelligence, Pulmonary nodule, Lung neoplasms, Adenocarcinoma, Invasive subtypes

## Abstract

**背景与目的:**

肺癌是国内外致死率最高的恶性肿瘤，肺结节的精确检测是降低肺癌死亡率的关键。人工智能辅助诊断系统在肺结节检测、良恶性鉴别和浸润亚型诊断等领域发展迅速，对其效能进行验证是促进其应用于临床的前提。本研究旨在评估人工智能辅助诊断系统预测肺结节早期肺腺癌浸润亚型的效能。

**方法:**

回顾性分析2016年1月1日-2021年12月31日期间兰州大学第二医院收治的223例肺结节早期肺腺癌患者的临床资料，将早期肺腺癌分为浸润性腺癌组(*n*=170)和非浸润性腺癌组(*n*=53)，其中非浸润性腺癌组又分为微浸润性腺癌组(*n*=31)和浸润前病变组(*n*=22)。比较各组的恶性概率和影像特征等信息，分析其对早期肺腺癌浸润亚型的预测能力，并对人工智能辅助诊断早期肺腺癌浸润亚型定性诊断的结果与术后病理进行一致性分析。

**结果:**

早期肺腺癌不同浸润亚型肺结节的平均CT值(*P* < 0.001)、直径(*P* < 0.001)、体积(*P* < 0.001)、恶性概率(*P* < 0.001)、胸膜凹陷征(*P* < 0.001)、分叶征(*P* < 0.001)、毛刺征(*P* < 0.001)差异均有统计学意义; 随着早期肺腺癌不同浸润亚型浸润性增加，各组参数显性征象比例也逐渐升高; 在二分类问题上，人工智能辅助诊断系统定性诊断早期肺腺癌浸润亚型的敏感性、特异性及曲线下面积(area under the curve, AUC)分别为81.76%、92.45%和0.871; 在三分类问题上，人工智能辅助诊断系统定性诊断早期肺腺癌浸润亚型的准确率、召回率、F1分数及AUC分别为83.86%、85.03%、76.46%和0.879。

**结论:**

该人工智能辅助诊断系统对肺结节早期肺腺癌浸润亚型具有一定的预测价值，随着算法的优化和数据的完善或可为患者个体化治疗提供指导。

## Abstract (en)

**Background and objective:**

Lung cancer is the cancer with the highest mortality at home and abroad at present. The detection of lung nodules is a key step to reducing the mortality of lung cancer. Artificial intelligence-assisted diagnosis system presents as the state of the art in the area of nodule detection, differentiation between benign and malignant and diagnosis of invasive subtypes, however, a validation with clinical data is necessary for further application. Therefore, the aim of this study is to evaluate the effectiveness of artificial intelligence-assisted diagnosis system in predicting the invasive subtypes of early-stage lung adenocarcinoma appearing as pulmonary nodules.

**Methods:**

Clinical data of 223 patients with early-stage lung adenocarcinoma appearing as pulmonary nodules admitted to the Lanzhou University Second Hospital from January 1^st^, 2016 to December 31^th^, 2021 were retrospectively analyzed, which were divided into invasive adenocarcinoma group (*n*=170) and non-invasive adenocarcinoma group (*n*=53), and the non-invasive adenocarcinoma group was subdivided into minimally invasive adenocarcinoma group (*n*=31) and preinvasive lesions group (*n*=22). The malignant probability and imaging characteristics of each group were compared to analyze their predictive ability for the invasive subtypes of early-stage lung adenocarcinoma. The concordance between qualitative diagnostic results of artificial intelligence-assisted diagnosis of the invasive subtypes of early-stage lung adenocarcinoma and postoperative pathology was then analyzed.

**Results:**

In different invasive subtypes of early-stage lung adenocarcinoma, the mean CT value of pulmonary nodules (*P* < 0.001), diameter (*P* < 0.001), volume (*P* < 0.001), malignant probability (*P* < 0.001), pleural retraction sign (*P* < 0.001), lobulation (*P* < 0.001), spiculation (*P* < 0.001) were significantly different. At the same time, it was also found that with the increased invasiveness of different invasive subtypes of early-stage lung adenocarcinoma, the proportion of dominant signs of each group gradually increased. On the issue of binary classification, the sensitivity, specificity, and area under the curve (AUC) values of the artificial intelligence-assisted diagnosis system for the qualitative diagnosis of invasive subtypes of early-stage lung adenocarcinoma were 81.76%, 92.45% and 0.871 respectively. On the issue of three classification, the accuracy, recall rate, F1 score, and AUC values of the artificial intelligence-assisted diagnosis system for the qualitative diagnosis of invasive subtypes of early-stage lung adenocarcinoma were 83.86%, 85.03%, 76.46% and 0.879 respectively.

**Conclusion:**

Artificial intelligence-assisted diagnosis system could predict the invasive subtypes of early-stage lung adenocarcinoma appearing as pulmonary nodules, and has a certain predictive value. With the optimization of algorithms and the improvement of data, it may provide guidance for individualized treatment of patients.

肺癌是全球范围内癌症相关死亡的主要原因，居我国癌症死亡人数之首^[1]^。非小细胞肺癌占肺癌的80%-85%，其中约50%为肺腺癌^[2, 3]^。肺腺癌的早期表现为肺结节，随着胸部低剂量螺旋计算机断层扫描(computed tomography, CT)肺癌筛查的广泛开展，肺结节的检出率不断增加^[4, 5]^。肺结节在影像学上表现为直径≤3 cm的局灶性、类圆形、密度增高的实性或亚实性肺部阴影，可为孤立性或多发性，不伴肺不张、肺门淋巴结肿大和胸腔积液^[6]^。根据第五版胸部肿瘤世界卫生组织(World Health Organization, WHO)分类^[7]^：非典型腺瘤样增生(atypical adenomatous hyperplasia, AAH)与原位腺癌(adenocarcinoma *in situ*, AIS)并列，归类为前驱腺体病变，即浸润前病变; 腺癌分为微浸润性腺癌(minimally invasive adenocarcinoma, MIA)和浸润性腺癌(invasive adenocarcinoma, IAC)。已有研究^[8]^表明AIS完整切除之后，10年术后无复发生存率达100%，10年术后总体生存率为95.3%，进一步验证AIS为癌前病变，可通过完整切除治愈的特性。与AIS相同，手术切除的MIA被证实有相似的无复发生存率和总体生存率，但仍需证实浸润性成分是否以贴壁生长为主。对于IAC患者，5年总体生存率为49%-84%。此外，对于AAH和AIS，可采用密切随访或局部切除; MIA虽然无淋巴结和血行转移，但可转化为IAC，可采用肺叶切除或亚叶切除; IAC采用肺叶切除加淋巴结清扫。因此，临床迫切需要准确诊断肺结节早期肺腺癌浸润亚型，从而决定随访管理、手术时机和手术方式、术后随访及预后评估等。

目前病理结果是肺癌诊断的金标准，但由于病理检查为侵入性操作，限制了其临床应用。肺癌肿瘤标志物及影像学检查广泛应用于临床，但部分标志物如癌胚抗原的特异性不高，易造成临床诊断的误差。影像学检查(如胸部X线、CT、磁共振成像等)对诊断具有一定价值，但不同浸润亚型的早期肺腺癌具有相似的影像特征，即使是经验丰富的影像科医生也难以区分。在医学图像分析领域，与严重依赖于手工特征选择的经典机器学习方法相反，深度学习方法在解决图像识别/分类等更复杂的任务时更有效^[9-12]^，如肿瘤分期，特别是分级、分类和转移检测方面^[13, 14]^。Avanzo等^[15]^指出，深度学习可以自动提取放射性特征，而非采用手工提取的特征，可极大提高模式识别系统的性能。常见的深度学习模型包括卷积神经网络(convolutional neural network, CNN)、残差神经网络、自编码器(auto-encoder, AE)等，其中最常用的是CNN，是一种非线性的多层特征学习模型^[16]^。本研究使用了基于人工智能的肺结节辅助诊断系统(以下简称人工智能系统)，该系统应用三维卷积神经网络(3D-convolutional neural network, 3D CNN)在内的多种算法进行各种图像处理任务，除了常规报告结节的直径、密度、体积、平均CT值、恶性概率等数据，该人工智能系统前期对10, 000多例源自国内知名肺部专科医院和三甲医院的肺结节(以磨玻璃结节和亚厘米结节为主)影像数据和病理结果反复训练与学习，应用其通过大数据训练所得的“经验”，预测肺结节早期肺腺癌的浸润亚型，从而提高医生诊断效率和准确率。本中心的前期工作已经证实其在肺结节的检出及良恶性鉴别方面有一定的诊断价值^[17]^，本研究旨在探讨人工智能辅助诊断系统在肺结节早期肺腺癌浸润亚型诊断中的应用价值。

## 1 资料与方法

### 1.1 研究对象

选取2016年1月1日-2021年12月31日兰州大学第二医院收治的肺结节患者。入组223例经术后病理证实为早期肺腺癌的肺结节，均经人工智能系统分析并给出腺癌定性诊断结果; 其中男性96例(43%)，平均年龄(56.0±9.6)岁; 女性127例(57%)，平均年龄(55.4±10.3)岁。根据肺腺癌不同浸润亚型分为三组：浸润前病变组(AAH和AIS)22例，男性6例、女性16例，平均年龄(51.5±7.3)岁; MIA组31例，男性16例、女性15例，平均年龄(52.2±9.7)岁; IAC组170例，男性74例，女性96例，平均年龄(56.9±10.0)岁。纳入标准：肺结节最大径≤3 cm; 术前2周内薄层(≤1.25 mm)CT影像，至少包含1枚非钙化结节; 经手术病理证实为肺腺癌并且人工智能辅助诊断系统对其进行了定性诊断。排除标准：CT检测前接受活检、放疗或化疗等操作或治疗; CT图像存在严重呼吸或运动伪影; 其他部位恶性肿瘤转移至肺。本研究已通过兰州大学第二医院伦理委员会审批，批准号：2021A-159。

### 1.2 研究方法

#### 1.2.1 人工智能肺结节辅助诊断系统检测

将符合纳入、排除标准的患者胸部CT的DICOM图像导入人工智能肺结节辅助诊断系统[点内(上海)生物科技有限公司]中。记录结节直径、体积、恶性概率、平均CT值以及腺癌定性诊断结果等信息。

#### 1.2.2 病理检查

标本均在兰州大学第二医院病理科完成病理检查，肺结节标本均经福尔马林固定，石蜡包埋切片，用特殊方法染色，最终病理诊断由2名主治医师以上职称的病理医师确定。其中，肺腺癌浸润亚型包括：AAH，即病灶上皮细胞轻中度不典型增生，无间质性炎症反应和纤维增生; AIS表现为肿瘤细胞沿肺泡贴壁式生长，无间质、血管或胸膜浸润; MIA以贴壁生长方式为主，表现为孤立性且浸润范围≤0.5 cm; IAC为间质、血管和胸膜受侵犯，浸润范围 > 0.5 cm的肺腺癌。

#### 1.2.3 腺癌分类

为了探讨人工智能系统对肺结节早期肺腺癌浸润亚型的鉴别能力，根据病理结果将腺癌分为以下两类：①非浸润性腺癌(AAH+AIS+MIA) *vs* IAC; ②浸润前病变(AAH+AIS) *vs* MIA *vs* IAC。

### 1.3 统计学分析

采用SPSS 25.0统计软件对数据进行统计分析。符合正态分布的计量资料以均数±标准差(Mean±SD)表示，组间比较采取单因素方差分析; 不符合正态分布的计量资料以中位数(四分位数间距)[M (IQR)]表示，组间比较采取*Kruskal-Wallis* *H*检验; 计数资料采用例数(%)表示，组间比较采用*χ*^2^检验。此外，应用Python 3.9.7软件绘制混淆矩阵评价人工智能系统预测肺结节早期肺腺癌浸润亚型的三分类结果，指标包括准确率、召回率和F1得分，F1值越高，诊断效能越好。F1计算方式：2(精确度×召回率)/(精确度+召回率)。绘制受试者工作特征曲线(receiver operating characteristic curve, ROC)，金标准为病理结果，计算曲线下面积(area under the curve, AUC)。*P* < 0.05表明差异有统计学意义。

## 2 结果

### 2.1 不同浸润亚型间资料比较

三组患者在性别方面差异无统计学意义(*P*=0.204); 经单因素方差分析，早期肺腺癌不同浸润亚型肺结节的年龄(*P*=0.005)、直径(*P* < 0.001)、体积(*P* < 0.001)、平均CT值(*P* < 0.001)、恶性概率(*P* < 0.001)差异均有统计学意义; 见表 1。

**表 1:** 患者特征 Patient characteristics

Characteristic	AAH/AIS (*n*=22)	MIA (*n*=31)	IAC (*n*=170)	*P*
Age (yr)	51.5±7.3	52.2±9.7	56.9±10.0	0.005
Gender (Male: Female)	6:16	16:15	74:96	0.204
Mean CT value (HU)	-552.5±157.4	-416.5±193.4	-104.9±221.1	< 0.001
Diameter (cm)	0.9±0.3	1.0±0.4	1.9±0.5	< 0.001
Volume (mm^3^)	373.0 (295.0)	457.0 (800.0)	3, 828.0 (5, 559.0)	< 0.001
Malignant probability (%)	82.1±9.1	83.5±7.8	80.5±7.5	< 0.001
AAH: atypical adenomatous hyperplasia; AIS: adenocarcinoma *in situ*; MIA: minimally invasive adenocarcinoma; IAC: invasive adenocarcinoma.				

### 2.2 结节影像学特征与浸润亚型

在223例早期肺腺癌患者中，影像学表现为纯磨玻璃样结节(pure ground-glass nodule, pGGN)的有42例(18.8%)，部分实性结节(part-solid nodule, pSN)76例(34.1%)，实性结节(solid nodule, SN)105例(47.1%)。在pGGN中，AAH/AIS占比最高，为18例，其次是IAC和MIA，分别为13例和11例。pSN中，IAC有52例，其次是MIA有20例。SN全部为IAC，而无AAH、AIS及MIA; 见表 2。同时本研究还发现随着肺腺癌不同浸润亚型浸润性增加，各组显性征象比例也逐渐升高; 见表 3。

**表 2:** 结节类型与浸润亚型的关系[*n*(%)] The relationship between nodule type and invasive subtypes [*n*(%)]

Density	Final pathology			Total
	AAH/AIS	MIA	IAC	
pGGN	18 (81.8)	11 (35.5)	13 (7.6)	42
pSN	4 (18.2)	20 (64.5)	52 (30.6)	76
SN	0 (0.0)	0 (0.0)	105 (61.8)	105
Total	22	31	170	223
pGGN: pure ground-glass nodule; pSN: part-solid nodule; SN: solid nodule.				

**表 3:** 影像学特征与浸润亚型的关系[*n*(%)] The relationship between imaging characteristics and invasive subtypes [*n*(%)]

Radiological sign	Final pathology			*P*	Density			*P*
	AAH/AIS (*n*=22)	MIA (*n*=31)	IAC (*n*=170)		pGGN (*n*=42)	pSN (*n*=76)	SN (*n*=105)	
Vascular penetration	19 (86.4)	27 (87.1)	156 (91.8)	0.477	37 (88.1)	70 (92.1)	95 (90.5)	0.754
Pleural retraction	2 (9.1)	6 (19.4)	113 (66.5)	< 0.001	4 (9.5)	33 (43.4)	84 (80.0)	< 0.001
Lobulation	2 (9.1)	8 (25.8)	134 (78.8)	< 0.001	6 (14.3)	48 (63.2)	90 (85.7)	< 0.001
Spiculation	1 (4.5)	3 (9.7)	116 (68.2)	< 0.001	2 (4.8)	40 (52.6)	78 (74.3)	< 0.001

### 2.3 诊断性能评估

图 1所示为人工智能系统对于早期肺腺癌浸润亚型的定性诊断结果与相应病理。将早期肺腺癌浸润亚型处理成非浸润性腺癌(AAH+AIS+MIA)和IAC的二分类问题时，人工智能系统的敏感性、特异性和AUC分别为81.76%、92.45%和0.871，ROC曲线见图 2。而将早期肺腺癌浸润亚型处理成浸润前病变(AAH+AIS)、MIA和IAC的三分类问题时，绘制混淆矩阵评价人工智能系统的诊断效能，其准确率、召回率和F1分数分别为83.86%、85.03%和76.46%，见图 3; 人工智能系统的AUC为0.879，ROC曲线见图 4。

**图 1:**
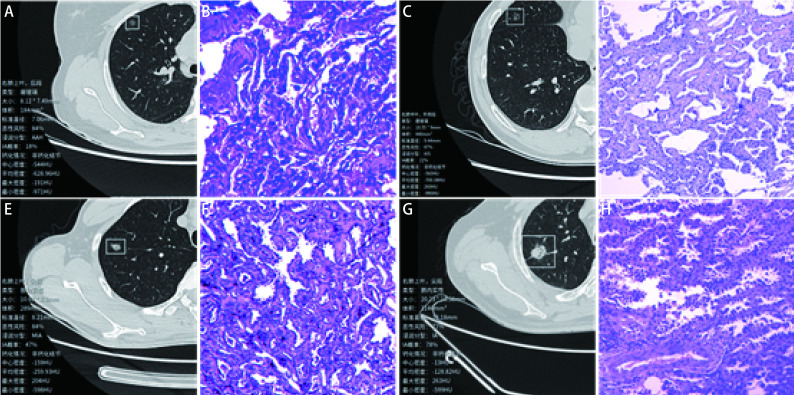
人工智能系统定性诊断早期肺腺癌浸润亚型与相应病理。A、B：不典型腺瘤样增生(图 1B，HE，×200); C、D：原位腺癌(图 1D，HE，×100); E、F：微浸润性腺癌(图 1F，HE，×200); G、H：浸润性腺癌(图 1H，HE，×200)。 Artificial intelligence system for qualitative diagnosis of the invasive subtypes of early-stage lung adenocarcinoma and the corresponding pathology. A, B: atypical adenomatous hyperplasia (Fig 1B, HE, ×200); C, D: adenocarcinoma *in situ* (Fig 1D, HE, ×100); E, F: minimally invasive adenocarcinoma (Fig 1F, HE, ×200); G, H: invasive adenocarcinoma (Fig 1H, HE, ×200).

**图 2:**
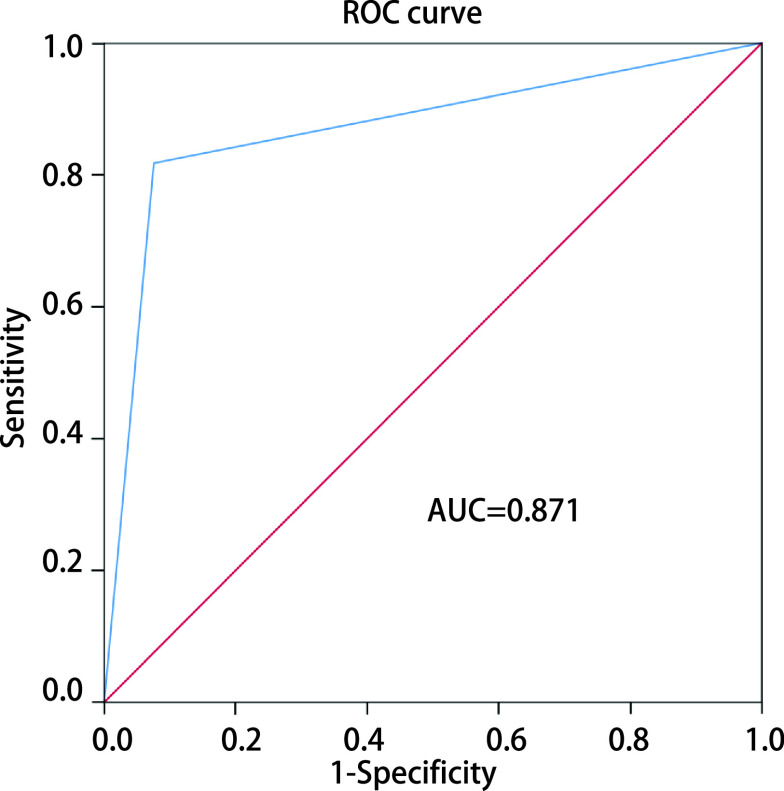
二分类结果ROC曲线 ROC curve for two-class result. ROC: receiver operating characteristic curve.

**图 3:**
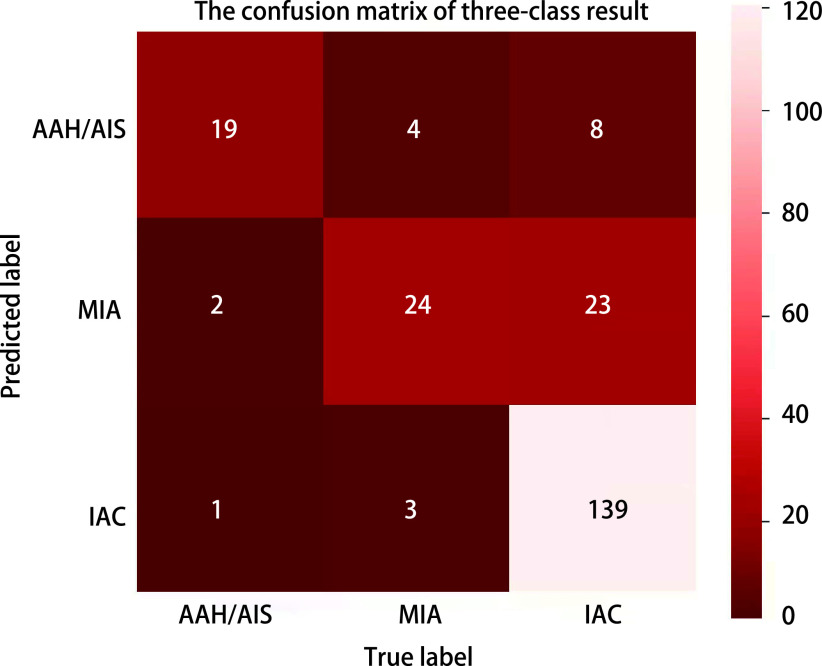
三分类结果的混淆矩阵 The confusion matrix of three-class result

**图 4:**
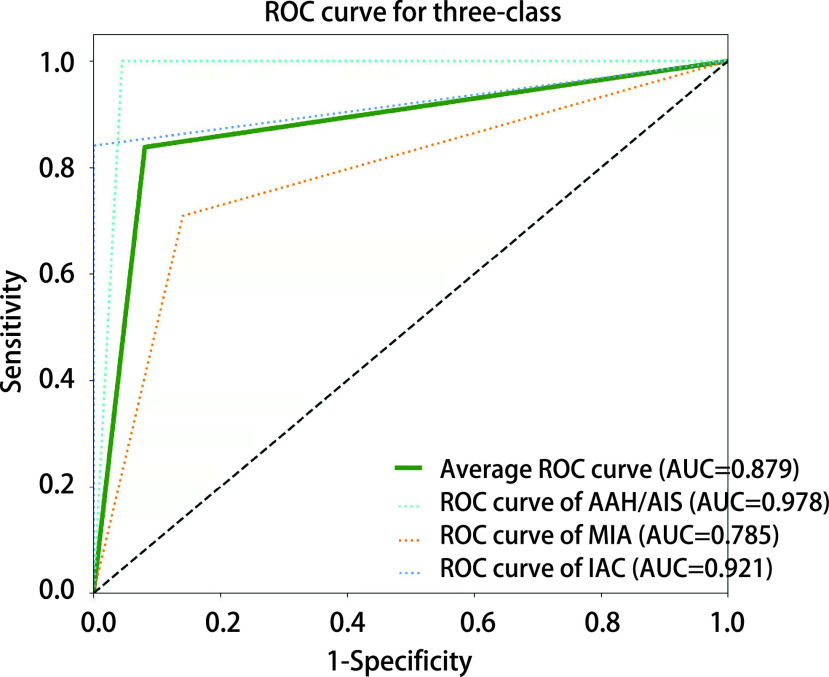
三分类结果的ROC曲线 ROC curve for three-class result

## 3 讨论

CT薄层扫描技术在肺结节的检测、诊断分类、治疗方案的制定、疗效评价及预后评估等方面发挥着重要的作用^[18, 19]^。临床工作中影像科医生常使用几种形态学特征来评估胸部CT肺结节的恶性特征，包括结节大小、边缘、轮廓和内部特征，而蕴含在数字化图像中的大量信息没有被利用。深度学习是由数据驱动的“端到端”特征学习方法，无需繁琐和费力的人工处理数据，通过自动检测原始数据(如像素、字符等)的特征信息并进行分类，然后按照非线性模块化的方式学习到多个层次上的特征，尤其适用于解决图像分类问题^[16, 20]^。目前已有研究^[21-24]^表明深度学习在肿瘤病灶良恶性鉴别、浸润亚型诊断、基因表达及预后评估方面表现出较好的应用前景。随着人工智能的发展，对于早期肺腺癌而言，通过人工智能产生的算法，协助外科医师选择合适的治疗方案将是未来的发展方向。

本研究首先收集术前患者的胸部CT影像，导入人工智能系统完成自动读片，检测肺结节影像数据并预测恶性概率和浸润亚型。判断恶性结节最主要的影像学特征首先是其直径，随着结节直径增大，浸润程度增加。本研究结果显示，IAC的直径明显大于非浸润性腺癌，IAC组与其他各组均具有显著差异(*P* < 0.05)，且直径为12.97 mm时诊断IAC的灵敏度(82.9%)和特异度(84.9%)最高。由于肺结节并非球形，无法根据结节直径准确估计结节大小，本研究使用基于人工智能的全自动测量方法或可更加准确地评估肺结节体积，研究表明，IAC的体积显著高于非浸润性腺癌(*P* < 0.05)，AUC为0.889，提示该测量方法具有较好的诊断效能。此外，本研究中使用基于深度学习的人工智能系统，对肺结节恶性概率进行分析，发现恶性概率在非浸润性腺癌组显著高于IAC组(*P* < 0.05)，AUC为0.724，这可能与提供给人工智能系统训练与学习的肺结节影像数据主要为磨玻璃结节有关。以上数据表明该人工智能系统能较为准确地区分肺结节的浸润亚型，可为临床制定诊疗方案提供有价值参考。

肺结节病理学从AAH、AIS、MIA到IAC是一个渐进发展的过程，影像学从pGGN、pSN到SN的发展也是一个进展的过程。当浸润程度较小时，肿瘤细胞大部分沿着肺泡壁贴壁式生长，无明显外侵倾向，多呈现为近似圆形，CT表现为磨玻璃样病变; 当浸润程度增大时，肿瘤细胞向各个方向生长速度不一，并对周围组织有一定的牵拉，从而产生不规则的形状，CT表现为实性结节。根据本组数据，浸润前病变中，pGGN占比最高，为18例，其次是pSN和SN，分别为4例和0例。在MIA中，pSN共20例，其次是pGGN 11例。在IAC中，主要为SN和pSN，分别为105例和52例，与前文所述的肿瘤发生发展过程相吻合，与人工智能系统定量参数相结合，可进一步提高术前诊断的准确率，对手术时机的选择及随访时间的判断具有重要意义。

然而利用上述传统定性定量参数对早期肺腺癌浸润亚型进行诊断并不能体现出人工智能的诊断优势，人工智能的优势在于识别一些人眼无法辨认的重要信息，从而对特定任务表现出较好的预测性能。本研究使用基于深度学习的人工智能系统在早期肺腺癌定性诊断的二分类问题上取得了良好的分类效果，其AUC为0.871，并获得了较高的灵敏度(81.76%)和特异度(92.45%)，先前已有学者对基于深度学习的人工智能系统辅助诊断早期肺腺癌浸润亚型进行相应的研究^[25-27]^。Wang等^[26]^基于编解码器框架实现肺结节自动检测，进一步结合浅层神经网络区分表现为GNN的IAC与非浸润性腺癌，该诊断方法的准确率为84.0%，敏感性为88.5%，AUC为0.892。Kim等^[27]^使用基于CT图像的2.5D DenseNet模型鉴别GGN中的IAC，结果表明2.5D DenseNet的Brier分数(0.122)优于基于大小的逻辑回归模型(0.198)，具有与影像科医生相当甚至略高(AUC: 0.921 *vs* 0.848-0.910)的诊断效能。此外，基于深度学习的人工智能系统在早期肺腺癌定性诊断的三分类问题已有不少文献报道，Zhao等^[28]^提出一种从低剂量CT图像自动分类亚厘米肺腺癌浸润程度的深度学习方法，该方法融合3D CNN和多任务学习算法的协同作用以分类亚厘米肺腺癌浸润亚型。结果显示，深度学习方法的准确率为64.1%，F1_AVG_达63.3%，马修斯系数MCC为0.407。Yu等^[29]^使用基于三维视觉几何群(3D visual geometry group, 3D VGG)的多任务深度学习网络，对CT引导下经皮穿刺定位的GNN浸润亚型进行分析，准确率为64.9%，F1_AVG_达65.1%，马修斯系数MCC为0.472。在临床工作中，如何发挥人工智能系统预测肺结节早期肺腺癌浸润亚型的价值，提升医生诊断准确性从而制定个体化的诊疗方案？这是一个值得思考的问题。Deng等^[22]^将基于三维深度学习辅助诊断系统(SSNet)的预测结果与6名不同年资的胸外科和影像科医生的判断结果进行比较，评估SSNet在预测GGN浸润程度时的效能。在浸润亚型分类中，SSNet取得了比6名医生更好的表现。在SSNet的帮助下，医生的AUC在三分类亚型鉴别中显著提高，AUC由0.844提升至0.852。上述研究表明基于深度学习的人工智能系统可潜在地提高临床医生判别GGN浸润亚型的诊断效能，在一定程度上可以对早期肺腺癌浸润亚型做出预测。此前，注意力机制在ImageNet上良好的性能表现引起了国内外学者的兴趣，SENet^[30]^将通道注意力机制引入2D图像分类，成为ImageNet 2017的冠军模型。Ni等^[31]^将空间注意力机制和通道注意力机制相结合，构建基于Attention-v1的3D CNN评估GGN的浸润性，其AUC达到最佳为0.926，比残差神经网络和随机森林模型性能更优，提示基于Attention-v1的3D CNN可进一步提高分类的性能。本研究使用基于注意力机制的3D CNN模型对输入的每个部分赋予不同的权重，抽取出更加关键及重要的信息，提高任务处理的效率和准确性，通过构建混淆矩阵评价三分类结果，其准确率、召回率、F1分数和AUC分别为83.86%、85.03%、76.46%和0.879。上述研究表明基于深度学习算法的人工智能系统可作为一种非侵入性工具在术前对早期肺腺癌的浸润亚型进行初步分析，在术中与冰冻切片结合或可指导手术切除范围的制定，并在早期和随访期间管理疾病。

本研究尚存在一定的局限性：①本研究为单中心回顾性研究，且仅纳入手术切除的原发性肺腺癌，未纳入非手术活检的患者，存在一定的选择性偏倚; ②本研究样本量较小，且不同浸润亚型的腺癌数量也不平衡，需要对大量病例进行进一步的前瞻性研究，以验证目前的预测模型; ③本研究仅纳入人工智能系统定性诊断的肺结节早期肺腺癌，然而该系统仍对21.5%的腺癌尚无把握进行定性诊断，需要对其进行算法优化或改进。

综上所述，人工智能系统通过术前定性诊断肺结节早期肺腺癌浸润亚型，与术后病理诊断相互验证，随着样本量的不断增加，人工智能系统诊断早期肺腺癌浸润亚型的准确率将不断提高，结合影像学征象的动态变化，进一步论证CT影像肿瘤进展与组织学增殖概念相吻合，为腺癌的发生发展理论提供佐证; 随着相关训练资料的不断完善以及深度学习网络算法的改进，该人工智能系统未来或可对肺结节早期肺腺癌浸润亚型进行更加全面的分析预测，从而为患者个体化治疗提供指导。
